# Obesity-and lipid-related indices as a predictor of hypertension in Mid-aged and Elderly Chinese: A Cross-sectional Study

**DOI:** 10.21203/rs.3.rs-2708175/v1

**Published:** 2023-03-28

**Authors:** Jiaofeng Gui, Yuqing Li, Haiyang Liu, Lei-lei Guo, Jinlong Li, Yunxiao Lei, Xiaoping Li, Lu Sun, Liu Yang, Ting Yuan, Congzhi Wang, Dongmei Zhang, Jing Li, Mingming Liu, Ying Hua, Lin Zhang

**Affiliations:** Wannan Medical College; Wannan Medical College; Wannan Medical College; Jinzhou Medical University; North China University of Science and Technology; Wannan Medical College; Wannan Medical College; Wannan Medical College; Wannan Medical College; Wannan Medical College; Wannan Medical College; Wannan Medical College; Wannan Medical College; Wannan Medical College; Wannan Medical College; Wannan Medical College

**Keywords:** hypertension, lipids, obesity, middle-aged and elderly Chinese

## Abstract

**Objective:**

Middle-aged and elderly people in China probably suffer from hypertension. There is a close relationship between obesity-and lipid-related index and hypertension, which is recognized by recent studies. However, these studies have not systematically compared the relationship between the two. We aim to find the most effective obesity-and lipid-related index for predicting hypertension.

**Method:**

A total of 9488 middle-aged and elderly people in China participated in this study. In this study, the subjects were divided into male and female groups by the definition of the 2018 Chinese Guidelines for Prevention and Treatment of Hypertension. Searching for the best predictors among 13 obesity-and lipid-related indicators through binary logistic regression analyses and receiver operator curve (ROC). These 13 indicators are body mass index (BMI), waist circumference (WC), waist-height ratio (WHtR), conicity index (CI), visceral adiposity index (VAI), Chinese visceral adiposity index (CVAI), lipid accumulation product (LAP), a body shape index (ABSI), body roundness index (BRI), triglyceride glucose index (TyG-index) and its correlation index (TyG-BMI, TyG-WC, TyG-WHtR)

**Results:**

After adjusting bias, all 13 indexes are risk factors for hypertension. In ROC curve analysis, thirteen obesity-and lipid-related factors can predict the occurrence of hypertension. Among them, CVAI has the best prediction effect (male: AUC = 0.660, female: AUC = 0.699). AUC for WHtR was equal to that for BRI and TyG - WHtR in identifying hypertension in male. Similarly, AUC of TyG-BMI and BMI were the same. In females, AUC for WHtR and BRI were the same when predicting hypertension. AUC of ABSI was much lower than other test indexes.

**Conclusion:**

In predicting hypertension, thirteen obesity-and lipid-related factors are effective. In addition, in males and females, CVAI is the best indicator to indicate hypertension. TyG-WHtR, WHtR, and BRI performed well in predicting metabolic syndrome in both males and females. ABSI has a poor ability to predict hypertension.

## Introduction

Hypertension is the most common risk factor for cardiovascular disease among middle-aged and elderly people in China. Hypertension is defined as systolic blood pressure (SBP) ≥ 140mmhg, and/or diastolic blood pressure ≤ 90mmhg, and/or taking antihypertensive drugs within two weeks[1]. In a study of 977 participants, the incidence of hypertension was as high as 29.49%, while only 43.2% of hypertension patients are fully aware of their overall disease condition[2]. According to a national survey on high blood pressure among adults in China conducted since 2012, the prevalence of high blood pressure among adults in China is about 23.2%[3]. However, the prevalence rate of hypertension the middle-aged and elderly people in China is as high as 56.1%, and the prevalence rate in male is higher than that in female[4]. With the aging of the population and increasing obesity, the number of people with high blood pressure has increased, and it is estimated that one-third of the world’s population will suffer from high blood pressure by 2025[5].

Some global disease studies[6–8] show that high blood pressure is the most common risk factor for heart disease and a major risk factor for death and disability worldwide. Hypertension is closely associated with the risk of certain tumors[9]. Kaneko, H[10] study shows that stage 2 and above hypertension can lead to an increased risk of colorectal cancer. In addition, hypertension is an important influencing factor of Alzheimer’s disease[11]. The decline of the metabolic ability of middle-aged and elderly people is accompanied by various basic diseases, which makes hypertension cause a greater risk of disability and death. Therefore, this study aims to find a more effective index to predict the occurrence of hypertension. Population and social development factors (age, gender, education, and region) are closely related to the prevalence of hypertension in China middle-aged and elderly people[12–14]. Smoking and drinking are significantly related to the incidence of hypertension[15]. At the same time, You, Y[16] research shows that strenuous physical activity does a good job of preventing high blood pressure.

It has been confirmed that obesity-and lipid-related indices are related to hypertension[17–19]. The correlation strength between them is the main research direction at present. A recent cross-sectional study conducted with 2740 patients by Tang, N[20] found that overweight (BMI 24.0–27.9 kg/m^2^) or obese (BMI ≥ 28.0 kg/m^2^) subjects have a higher risk of hypertension than normal body mass index. Another cross-sectional study performed for the 22894 participants by Sun, J.Y[21] found that WC indicated best in predicting hypertension. Most cross-sectional studies were designed to explore the intensity of the association between a single indicator and hypertension. There is no comprehensive comparison of obesity- and lipid-related indicators in the prediction of high blood pressure. At the same time, there are differences among various ethnic groups, while research on Asian populations is still blank. Therefore, it is necessary to explore obesity-and lipid-related indicators to predict the ability of hypertension in middle-aged and elderly people in China.

Data from this cross-sectional study were collected from community residents over 45 years of age in China. We compared the correlation strength between obesity-and lipid-related indicators and hypertension through the data of representative samples, aiming to find the most appropriate prediction indices of hypertension.

## Materials And Methods

### Participants

Participants in this cross-sectional study were China community residents over the age of 45. All participants were from China Health and Retirement Longitudinal Study (CHARLS). CHARLS is a nationally representative longitudinal survey. Every two years, CHARLS conducted computer-assisted personal interviews (CAPI) and structured questionnaires with participants. In the survey, 17,284 participants were 45 years of age or older. CHARLS collected data from 2011, 2013 and 2015, and we used the data of 2011. We excluded participants who were not followed up, as well as any standard individuals without data on age, sex, education, smoking history, activity participation, regular exercise, and chronic disease. The number of people who completed both baselines without hypertension symptoms was 9488.

### Hypertension symptom

According to the 2018 Chinese Guidelines for Prevention and Treatment of Hypertension[22], Clinical systolic blood pressure≥ 140mmHg or diastolic blood pressure≥ 90 mmHg was defined as hypertension. Blood pressure is usually measured with an international standardized upper arm medical electronic sphygmomanometer or a mercury sphygmomanometer that meets measurement standards (at least 5 minutes of sitting in a quiet environment). Diagnosis of hypertension is divided into three categories:

1. Clinic systolic BP≥ 140 mmHg or diastolic BP≥ 90 mmHg (antihypertensive drugs were never used in three different visits).
2. Clinic systolic BP < 140/90 mmHg (having the hypertensive history and currently taking anti-hypertensive medication).

### Covariates

In this study, we divided the participants into a male group and a female group. At the same time, we regard age, education level, marital status, current residence, current smoking, drinking, chronic diseases, participation in activities, and regular exercise as covariates of this study. We counted 14 chronic diseases and grouped them according to the number of diseases, which is the same as our previous studies[23, 24]. The nine covariates are shown below.

1. Age: 1) below 45–54, 2) 55–64, 3) 65–74, 4) above 75.
2. Education level: 1) illiterate, 2) less than elementary school,3) high school, 4) above vocational school.
3. Marital status: 1) the single (divorced, and never married, widowed, or separated), 2) married.
4. Current residence: 1) rural, 2) urban.
5. Current smoking: 1) current smokers, 2) former smokers, 3) never smokers.
6. Alcohol drinking: 1) never drinker, 2) less than once a month, 3) more than once a month.
7. Taking activities: 1) yes, 2) no.
8. Having regular exercises: 1) no physical exercise, 2) less than regular physical exercises, 3) regular physical exercises.
9. Chronic diseases: 1) 0, 2) 1–3, 3) 4–16.

These standards are also applied in our previous research[23, 25–32].

### Measurements

Waist circumference (WC) is the circumference of the line connecting the lowest point of the rib to the midpoint of the upper edge of the iliac crest before the end of expiratory breath[33]. It should be noted that the other 12 indicators need to be calculated. At the same time, some indicators need to be invasive to obtain TG and HDL. Body mass index (BMI) is the value of weight (kg) divided by the square of height (m)[34]. Waist height ratio (WHtR) is the ratio of WC (m) to height (m)[35]. The calculation of visceral adiposity index (VAI) differs between males and females but is based on WC, BMI, TG, and HDL[36]. Chinese visceral adiposity index (CVAI) is based on VAI, according to the physical characteristics of China people to develop indicators[37]. Similarly, the calculation method of lipid accumulation product (LAP) is different due to gender differences[38]. A body shape index (ABSI) is obtained by WC, BMI, and height[39]. Body roundness index (BRI) is obtained through WC and height[40]. Conicity index (CI) is obtained through WC, weight, and height[41]. Triglyceride glucose index (TyG-index) is a lipid index calculated from TG and glucose[42]. At the same time, TyG index combined with BMI, WC, and WHtR constitutes TyG-BMI, TyG-WC, and TyG-WHtR[43–45]. We have listed the calculation formula for the 12 indicators below:

1. BMI = Weight/Height^2^
2. WHtR = WC/Height
3. Males: VAI = WC/(1.88×BMI + 39.68) ×TG/1.03×1.31/HDL
  Females: VAI = WC/(1.89×BMI + 36.58) ×TG/0.81×1.52/HDL
4. $\text{ABSI}=\frac{WC}{{H\text{eight}}^{\frac{1}{2}}\times {\text{BMI}}^{\frac{2}{3}}}$
5. $\text{BRI}=364.2-365.5\sqrt{1-\left(\frac{WC÷{\left(2\pi \right)}^{2}}{{\left(0.5\times \text{Height}\right)}^{2}}\right)}$
6. Males: LAP = TG ×(WC-65)
  Females: LAP = TG ×(WC-58)
7. $\text{CI}=\text{WC}/\sqrt[{0.19}]{\text{Weight}/\text{Height}}$
8. Males: CVAI=−267.93–16.32×HDL-C + 0.03×BMI + 22.00×Log_10_TG + 0.68×age + 4.00×WC
  Females: CVAI=−187.32–11.66×HDL-C + 4.32×BMI + 39.76×Log_10_TG + 1.71×age + 1.12×WC
9. TyG index = Ln(TG ×glucose/2)
10. TyG-BMI = TyG×BMI
11. TyG-WC = TyG×WC
12. TyG -WHtR = TyG×WHtR

### Statistical analysis

In this study, SPSS version 25.0 (IBM SPSS, Armonk, NY, USA) was used for statistical analysis of all obtained sample data. The chi-square test was applied to classified variables and the t-test was applied to continuous variables to determine the strength of differences between the variables. The odds ratio (OR) of obesity-related and lipid-related indices to hypertension before and after adjustment of the bias was calculated. These biases included age, education level, marital status, current residence, current smoking, alcohol drinking, taking activities, regular exercise, and chronic diseases. The ability of these indicators to predict hypertension was compared by receiver operator curve (ROC).

## Results

Table 1 shows the baseline characteristics of participants according to gender difference. The number of participants was 9488. 45.89% were male, and 54.11% were female. 12.45% were single. 92.40% were living in rural. 24.69% of males do not smoke, 92.19% of females do not smoke, 13.71% of males have never received education, while 42.56% of females have never received education. Among them, 24.69% of males have never smoked, and 92.19% of females have never smoked. 58.45% of males are smoking, and 5.96% of females are smoking. There are significant differences in education level, smoking and drinking between males and females. These 13 obesity-and lipid-related indices are different between male and female groups (P < 0.05).

Table 2 shows the baseline characteristics of men and women with and without hypertension, respectively. 9488 participants were divided into two groups. In 4354 males, 1742(40.01%) males had hypertension and 2612(59.99%) males had no hypertension. In 5134 females, 2162(42.11%) females suffered from hypertension, and 2972(57.89%) females did not suffer from hypertension. There were significant differences in age and chronic disease between hypertensive and non-hypertensive patients, as well as differences in 13 indicators between the two groups. (P < 0.05).

Table 3 shows that hypertension increased progressively with unit increases in obesity- and lipid-related indices in both sexes. For instance, in men, a unit increase in WC was associated with 1.062- fold increased odds of elevated hypertension (OR: 1.062; 95% CI: 1.054–1.070), and a unit increase in BMI was associated with a 1.179-fold increase in odds of elevated hypertension (OR: 1.179; 95% CI: 1.154–1.205). In women, a unit increase in WC was associated with a 1.051- fold increase in odds of elevated hypertension (OR: 1.051; 95% CI: 1.044–1.057), and a unit increase in BMI was associated with 1.138-fold increased odds of elevated hypertension (OR: 1.138; 95% CI: 1.119–1.158).

**Figure 1:**
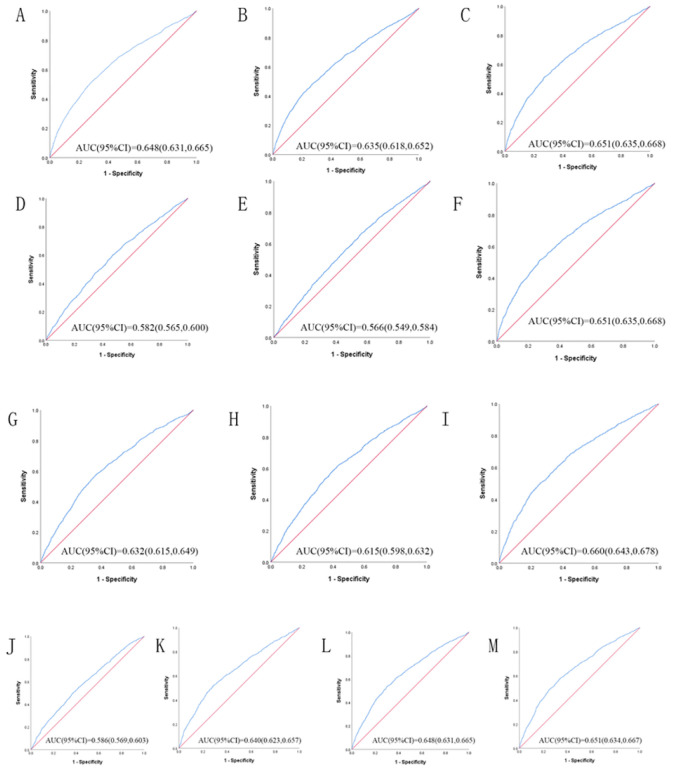
The ROC curves of each indicator in the prediction of MetS risk in male. (A) = WC, (B) = BMI, (C) = WHtR, (D) = VAI, (E) = ABSI, (F) = BRI, (G) = LAP, (H)= CI, (I) = CVAI, (J) = TyG-index, (K) = TyG-BMI, (L) = TyG-WC, (M) = TyG-WHtR.

**Figure 2:**
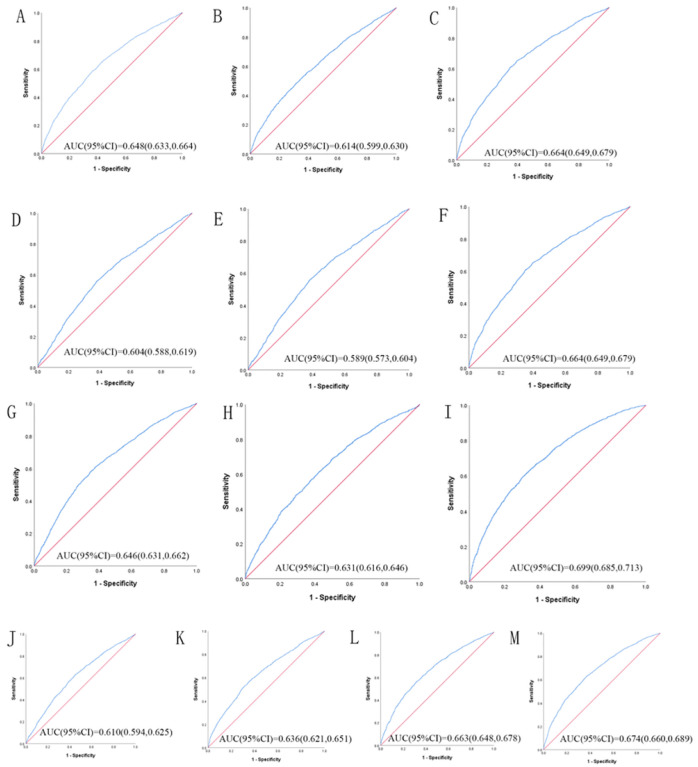
The ROC curves of each indicator in the prediction of MetS risk in female. (A) = WC, (B) = BMI, (C) = WHtR, (D) = VAI, (E) = ABSI, (F) = BRI, (G) = LAP, (H) = CI, (I) = CVAI, (J) = TyG-index, (K) = TyG-BMI, (L) = TyG-WC, (M) = TyG-WHtR.

Table 4 shows the ROC analysis between the 13 indicators and hypertension. Figure 1 and Fig. 2 show the ROC curve and AUC for males and females, respectively. In male, the largest AUC was observed for the CVAI (AUC = 0.660, 95%CI = 0.643–0.676 and optimal cut-off = 111.142). The prediabetes predictive values were similar for the WHtR (AUC = 0.651, 95%CI = 0.635–0.668, and optimal cut-off = 0.534), BRI (AUC = 0.651, 95%CI = 0.635–0.668, and optimal cut-off = 4.013), and TyG -WHtR (AUC = 0.651, 95%CI = 0.634–0.667, and optimal cut-off = 4.525). In women, the largest AUC was CVAI (AUC = 0.699, 95%CI = 0.685–0.713, and optimal cut-off = 113.022). The prediabetes predictive values were similar for the TyG -WHtR (AUC = 0.674, 95%CI = 0.660–0.689, and optimal cut-off = 4.924), WHtR (AUC = 0.664, 95%CI = 0.649–0.679, and optimal cut-off = 0.567), and BRI (AUC = 0.664, 95%CI = 0.649–0.679, and optimal cut-off = 4.697). It should be noted that these 13 indicators have significant significance for predicting the incidence of hypertension in both men and women (*P* < 0.001)

## Discussion

According to the 2018 Chinese Guidelines for Prevention and Treatment of Hypertension[22], This research predicts the risk index of hypertension in middle-aged and elderly people in China by the 13 obesity-related indexes. It is a novel one. We know that before this, there was no research paper to evaluate the most suitable index to predict the risk of hypertension by comparing 13 obesity and lipid-related index. This study included 9488 male and female participants over 45 years old. Among the 9488 subjects, the proportion of hypertension was 41.15%. Among the 4354 males, 1742(40.01%) and 5134 females, 2162(42.11%) suffered from hypertension. Based on the entire country’s investigation[3], the prevalence of high blood pressure among adults in China is about 23.2%, among which the prevalence of hypertension increases with age, the prevalence of hypertension in the population aged 65 and over is over 55%, and the prevalence rate in men is higher than that in women. Therefore, it is necessary to find a more suitable obesity-and lipid-related index to predict the incidence of hypertension in middle-aged and elderly people in China.

Table 3 shows that the ABSI is significantly related to hypertension in men, after adjusting the individual characteristics, but it is not statistically significant in women. Some studies [37, 46–48] show that ABSI is lower than other indicators in relation to hypertension, which is consistent with the results of this study. It is worth noting that the data in Table 4 shows that ABSI still has reference significance in predicting the incidence of hypertension among middle-aged and elderly people in China. However, ABSI’s prediction ability is far lower than the other 12 indexes. Cheung, Y.B[47] study showed that compared with WC and BMI, ABSI was less associated with middle-aged and elderly morbidity. Because the deviation of ABSI from its average value is low, ABSI is not significant in predicting the incidence of hypertension[49]. After adjusting the individual characteristics, the odds ratio (OR) and 95% CI of various obesity- and lipid-related indices to hypertension were calculated, and all reached statistical significance (*P* < 0.05).

ROC analysis of obesity- and lipid-related indices showed that the AUC of all indexes of male and female was statistical meaning (*P* < 0.05). Among the 13 indexes, CVAI, WHtR, BRI, and TyG -WHtR have received special attention.

In a cross-sectional study[50] of 14,573 participants, VAI was a better predictor of hypertension in both men and women. VAI proposed by Amato M.C can effectively evaluate visceral fat function[36]. A study conducted by Fiorentino, T.V[51] to test the risk factors related to the progression of hypertension in patients with prehypertension showed that VAI was an independent risk factor for the progression of hypertension. However, the significant overlap of confidence intervals of VAI did not mean that VAI was superior to other obesity indicators in predicting the development of hypertension. Notably, the combination of VAI and WC shows the highest predicted values for hypertension. This conclusion is consistent with the results of a cohort study[52] conducted in Chengdu, Sichuan Province, China. It can be analyzed for the following reasons: the difference of body fat distribution between different races is also obvious. According to the distribution law of body fat in Asian population, Xia, M. F. put forward the index of CVAI based on VAI to calculate the visceral fat area of China people[41]. In this study, compared with VAI, CVAI is more prominent in evaluating the prevalence of hypertension in middle-aged and elderly people in China.

A cohort study[53] of 10,304 Chinese adult residents showed that CVAI outperformed other measures of visceral obesity in predicting the incidence of hypertension in either men or women. A study by Li, B[54] has shown that CVAI is more effective in discriminating hypertension and prehypertension among the general population in China. Similarly, Lin, M[55], in a 2022 cohort of 2, 033 participants, showed that CVAI performed best in predicting hypertension. In this study, ROC analysis was performed on obesity-and lipid-related indicators, maximum AUC (male: AUC = 0.660, 95% CI = 0.643–0.676 and optimal cut-off = 111.142, female: AUC = 0.699, 95% CI = 0.685–0.713, and optimal cut-off = 113.022) was observed for the CVAI. CVAI showed the best ability to predict the occurrence of hypertension in both men and women. This conclusion has corresponded with the conclusion of the previous study.

WHtR and BRI have obvious advantages in forecasting the incidence of hypertension in middle-aged and elderly people[56]. In this study, WHtR and BRI are indicators for predicting the occurrence of hypertension, and their prediction ability is second only to CVAI. Lee, J. W[57] study showed that WHtR and WC were superior to BMI as screening tools in forecasting the incidence of hypertension in middle-aged and elderly Koreans. At the same time, A prospective cohort study[58] of 812 participants showed that WHtR had a significant advantage over WC, and BMI in predicting the development of hypertension. Similarly, in a cross-sectional study by Saeed, A. A[59], WHtR was the best predictor of hypertension among various anthropometric measures. These studies are basically consistent with the view of this paper that WHtR has a good performance in predicting the occurrence of hypertension.

Some obesity-and lipid-related indices provide a more reliable basis for predicting the occurrence of hypertension. In this study, the association strength between four lipid indexes, such as TyG index, TyG- body mass index, TyG-WC and TyG-WHtR, and hypertension was evaluated. In fact, a large number of studies[60–63] have shown that there is a significant relationship between TyG related factors and hypertension. In this study, the ability of WHtR (AUC = 0.651, 95% CI = 0.635–0.668) and TyG-WHtR (AUC = 0.651, 95%CI = 0.634–0.667) to predict the occurrence of hypertension in men was approximately the same. But in women, the prediction ability of TyG-WHtR (AUC = 0.674, 95%CI = 0.660–0.689) was superior to WHtR (AUC = 0.664, 95%CI = 0.649–0.679). Therefore, TyG-WHtR is more accurate than WHtR in predicting the occurrence of hypertension in the middle-aged and elderly population in China.

BRI has the sufficient discriminating ability to discriminate hypertension from the normotensive population[64]. Chang, Y study[65] of two new measures to identify high blood pressure reported a low association with ABSI and the strongest association with BRI. A study[66] that predicted risk factors for cardiovascular disease in China reported that BRI was superior to other indicators in its predictive power. It should be noted that BRI and WHTR did not show a significant difference in both men and women.

### Strengths and limitations of the study

This study has several advantages. This study was based on a nationwide cohort study of middle-aged and older community residents, with participants aged 45 years or older. It compared the effect of different obesity-and lipid-related indices hypertension and its components symptom. Previous studies used only a set of single indices to predict the Incidence of hypertension. It helped us to understand the different obesity-and lipid-related indices on the incidence of hypertension. There are several limitations to this study. Many participants were excluded due to missing data, and further studies should gather more complete data.

## Conclusions

After adjusting the bias factors, all 13 obesity-and lipid-related factors were independently related to hypertension and all presented as risk factors. All 13 obesity-and lipid-related factors show the ability to predict hypertension. CVAI is the indicator with the best prediction ability, while ABSI has the worst prediction ability for hypertension. Additionally, TyG-WHtR, WHtR, and BRI performed well in predicting hypertension in both males and females.

## Figures and Tables

**Table 1:** Characteristics of participants with full samples(N = 9488)

Variables	Male	Female	Total	*t/x* ^*2*^	*P*
N (%)	N (%)	N (%)			
N	4354(100)	5134 (100)	9488 (100)		
Age(years)				83.541	0.000
45–54	1274(29.26)	1922(37.44)	3196(33.68)		
55–64	1724(39.60)	1930(37.59)	3654(38.51)		
65–74	989(22.71)	903(17.59)	1892(19.94)		
≥75	367(8.43)	379(7.38)	746(7.86)		
Education				968.940	0.000
Illiterate	597(13.71)	2185(42.56)	2782(29.32)		
Less than elementary school	3195(73.38)	2604(50.72)	5799(61.12)		
High school	360(8.27)	253(4.93)	613(6.46)		
Above vocational school	202(4.64)	92(1.79)	294(3.10)		
Marital status				71.991	0.000
Single	406(9.32)	775(15.10)	1181(12.45)		
Married	3948(90.68)	4359(84.90)	8307(87.55)		
Current residence				0.750	0.387
Rural	4012(92.15)	4755(92.62)	8767(92.40)		
Urban	342(7.85)	379(7.38)	721(7.60)		
Current smoking				4521.237	0.000
No	1075(24.69)	4733(92.19)	5808(61.21)		
Former smoke	734(16.86)	95(1.85)	829(8.74)		
Current smoke	2545(58.45)	306(5.96)	2851(30.05)		
Alcohol drinking				2162.870	0.000
No	1920(44.10)	4516(87.96)	6436(67.83)		
Less than once a month	470(10.79)	255(4.97)	725(7.64)		
More than once a month	1964(45.11)	363(7.07)	2327(24.53)		
Taking activities				0.789	0.374
No	2138(49.10)	2568(50.02)	4706(49.60)		
Yes	2216(50.90)	2566(49.98)	4782(50.40)		
Having regular exercises				1.150	0.563
No exercise	2708(62.20)	3145(61.26)	5853(61.69)		
Less than exercises	814(18.70)	1001(19.50)	1815(19.13)		
Regular exercises	832(19.11)	988(19.24)	1820(19.18)		
Chronic diseases(counts)				20.702	0.000
0	1422(32.66)	1474(28.71)	2896(30.52)		
1–2	2161(49.63)	2623(51.09)	4784(50.42)		
3–14	771(17.71)	1037(20.20)	1808(19.06)		
WC	84.96 ±9.81	85.64 ±10.16	85.33 ±10.01	−3.319	0.001
BMI	22.96 ±3.64	23.99 ±4.05	23.51 ±3.90	−13.002	0.000
WHtR	0.52 ±0.06	0.56 ±0.07	0.54 ±0.07	−33.311	0.000
VAI	3.96 ±4.41	6.07 ±5.72	5.10 ±5.26	−20.256	0.000
ABSI	8.25 ±0.53	8.38 ± 0.64	8.32 ± 0.59	−10.650	0.000
BRI	3.78 ± 1.14	4.66 ± 1.46	4.26 ± 1.39	−33.183	0.000
LAP	30.87 ±33.31	43.74 ± 35.23	37.83 ± 34.95	−18.262	0.000
CI	1.27 ±0.08	1.30 ± 0.10	1.29 ± 0.09	−15.487	0.000
CVAI	95.98 ±47.50	107.11 ± 43.43	102.00 ± 45.68	−11.826	0.000
TyG index	8.62 ± 0.66	8.72 ± 0.63	8.68 ± 0.65	−7.594	0.000
TyG-BMI	198.67 ± 39.64	209.77 ± 41.67	204.68 ± 41.12	−13.285	0.000
TyG-WC	734.60 ± 117.99	748.68 ± 116.22	742.21 ± 117.24	−5.840	0.000
TyG -WHtR	4.48 ± 0.69	4.91 ± 0.76	4.71 ± 0.76	−28.240	0.000

WC: waist circumference; BMI: body mass index; WHtR: waist to height ratio; VAI: visceral adiposity index; ABSI: A body shape index; BRI: body roundness index; LAP: lipid accumulation product; CVAI: Chinese visceral adiposity index; CI: conicity index; TyG: triglyceride glucose; TyG-BMI: TyG related to BMI; TyG-WC: TyG related to WC; TyG-WHtR: TyG related to WHtR

**Table 2:** Baseline characteristics of the study participants with and without HTN by sex.

Variables	Male (N = 4354)		*t/x* ^*2*^	*P*	Female (N = 5134)		*t/x* ^*2*^	*P*
N (%)	With HTN N (%)	Without HTN N (%)			With HTN N (%)	Without HTN N (%)		
N	1742 (40.01)	2612 (59.99)			2162(42.11)	2972(57.89)		
Age(years)			93.439	0.000			367.339	0.000
45–54	392(22.50)	882(33.77)			1389(53.18)	533(30.60)		
55–64	686(39.38)	1038(39.74)			1085(41.54)	845(48.51)		
65–74	471(27.04)	518(19.83)			378(14.47)	525(30.14)		
≥ 75	193(11.08)	174(6.66)			120(4.59)	259(14.87)		
Education			15.299	0.002			36.816	0.001
Illiterate	256(14.70)	341(13.06)			1017(58.38)	1168(44.72)		
0Less than elementary school	1273(73.08)	1922(73.58)			1031(59.18)	1573(60.22)		
High school	117(6.72)	243(9.30)			80(4.59)	173(6.62)		
Above vocational school	96(5.51)	106(4.06)			34(1.95)	58(2.22)		
Marital status			20.502	0.000			81.926	0.000
Single	205(11.77)	201(7.70)			441(25.32)	334(12.79)		
Married	1537(88.23)	2411(92.30)			1721(98.79)	2638(101.00)		
Current residence			8.375	0.004			0.004	0.948
Rural	1580(90.70)	2432(93.11)			2003(114.98)	2752(105.36)		
Urban	162(9.30)	180(6.89)			159(9.13)	220(8.42)		
Current smoking			20.182	0.000			8.308	0.016
No	448(25.72)	627(24.00)			1966(112.86)	2767(105.93)		
Former smoke	340(19.52)	394(15.08)			48(2.76)	47(1.80)		
Current smoke	954(54.76)	1591(60.91)			148(8.50)	158(6.05)		
Alcohol drinking			2.205	0.332			9.221	0.010
No	792(45.46)	1128(43.19)			1935(111.08)	2581(98.81)		
Less than once a month	183(10.51)	287(10.99)			88(5.05)	167(6.39)		
More than once a month	767(44.03)	1197(45.83)			139(7.98)	224(8.58)		
Taking activities			0.817	0.366			4.476	0.034
No	870(49.94)	1268(48.55)			1044(59.93)	1524(58.35)		
Yes	872(50.06)	1344(51.45)			1118(64.18)	1448(55.44)		
Having regular exercises			1.444	0.486			7.158	0.028
No exercise	1091(62.63)	1617(61.91)			1368(78.53)	1777(68.03)		
Less than exercises	311(17.85)	503(19.26)			390(22.39)	611(23.39)		
Regular exercises	340(19.52)	492(18.84)			404(23.19)	584(22.36)		
Chronic diseases(counts)			348.618	0.000			487.229	0.000
0	344(19.75)	1078(41.27)			351(20.15)	1123(42.99)		
1–2	898(51.55)	1263(48.35)			1106(63.49)	1517(58.08)		
3–14	500(28.70)	271(10.38)			705(40.47)	332(12.71)		
WC	88.04 ± 10.39	82.91 ± 8.83	−16.937	0.000	88.70 ± 10.37	83.42 ± 9.40	−18.743	0.000
BMI	23.96 ± 3.98	22.29 ± 3.23	−14.647	0.000	24.89 ± 4.27	23.33 ± 3.74	−13.614	0.000
WHtR	0.54 ± 0.06	0.51 ± 0.05	−17.243	0.000	0.58 ± 0.07	0.54 ± 0.06	−20.751	0.000
VAI	4.56 ± 4.86	3.56 ± 4.03	−7.090	0.004	7.05 ± 6.50	5.36 ± 4.96	−10.126	0.000
ABSI	8.31 ± 0.54	8.21 ± 0.51	−6.013	0.000	8.49 ± 0.68	8.30 ± 0.59	−10.426	0.000
BRI	4.15 ± 1.23	3.53 ± 1.01	−17.337	0.000	5.15 ± 1.55	4.31 ± 1.27	−20.514	0.000
LAP	38.59 ± 38.65	25.71 ± 28.06	−11.964	0.000	53.08 ± 39.86	36.94 ± 29.65	−15.905	0.000
CI	1.29 ± 0.09	1.26 ± 0.08	−11.687	0.000	1.33 ± 0.10	1.28 ± 0.09	−15.807	0.000
CVAI	112.11 ± 49.52	85.22 ± 42.86	−18.507	0.000	124.49 ± 42.03	94.46 ± 39.92	−25.809	0.000
TyG index	8.74 ± 0.68	8.54 ± 0.63	−9.699	0.000	8.86 ± 0.65	8.62 ± 0.60	−13.051	0.000
TyG-BMI	210.27 ± 43.49	190.92 ± 34.77	−15.551	0.000	220.97 ± 43.92	201.63 ± 37.93	−16.485	0.000
TyG-WC	771.74 ± 125.33	709.82 ± 105.84	−16.976	0.000	787.09 ± 119.23	720.73 ± 105.55	−20.655	0.000
TyG -WHtR	4.70 ± 0.73	4.34 ± 0.62	−17.265	0.000	5.18 ± 0.78	4.71 ± 0.68	−22.339	0.000

WC: waist circumference; BMI: body mass index; WHtR: waist to height ratio; VAI: visceral adiposity index; ABSI: A body shape index; BRI: body roundness index; LAP: lipid accumulation product; CVAI: Chinese visceral adiposity index; CI: conicity index; TyG: triglyceride glucose; TyG-BMI: TyG related to BMI; TyG-WC: TyG related to WC; TyG-WHtR: TyG related to WHtR

**Table 4:** Area Under Curve for obesity-and lipid-related indices to detect HTN by sex

N = 4423	WC	BMI	WHtR	VAI	ABSI	BRI	LAP	CI	CVAI	TyG in
**Male**										
Area under curve	0.648	0.635	0.651	0.582	0.566	0.651	0.632	0.615	0.660	0.586
Std. Error	0.009	0.009	0.009	0.009	0.009	0.009	0.009	0.009	0.008	0.009
95%CI	0.631,0.665	0.618,0.652	0.635,0.668	0.565,0.600	0.549,0.584	0.635,0.668	0.615,0.649	0.598,0.632	0.643,0.676	0.569
*P*-value	0.000	0.000	0.000	0.000	0.000	0.000	0.000	0.000	0.000	0.000
Optimal cutoffs	87.250	23.877	0.534	2.516	8.120	4.013	22.955	1.272	111.142	8.647
J-Youden	0.233	0.212	0.240	0.131	0.106	0.240	0.215	0.189	0.245	0.129
Sensitivity (%)	51.90	48.10	51.40	59.90	66.90	51.40	57.90	61.50	50.30	49.80
Specificity (%)	71.40	73.10	72.60	53.20	43.70	72.60	63.60	57.40	74.20	63.10
(+) Likelihood ratio	1.815	1.788	1.876	1.280	1.188	1.876	1.591	1.444	1.950	1.350
(−) Likelihood ratio	0.674	0.710	0.669	0.754	0.757	0.669	0.662	0.671	0.670	0.796
**Female**										
Area under curve	0.648	0.614	0.664	0.604	0.589	0.664	0.646	0.631	0.699	0.610
Std. Error	0.008	0.008	0.008	0.008	0.008	0.008	0.008	0.008	0.007	0.008
95%CI	0.633,0.664	0.599,0.630	0.649,0.679	0.588,0.619	0.573,0.604	0.649,0.679	0.631,0.662	0.616,0.646	0.685,0.713	0.594
*P*-value	0.000	0.000	0.000	0.000	0.000	0.000	0.000	0.000	0.000	0.000
Optimal cutoffs	86.900	24.7859	0.567	4.711	8.380	4.697	37.406	1.309	113.022	8.624
J-Youden	0.224	0.170	0.257	0.177	0.148	0.257	0.234	0.191	0.293	0.179
Sensitivity (%)	58.50	48.20	60.40	55.80	55.20	60.40	58.30	56.80	59.60	62.90
Specificity (%)	63.90	68.80	65.30	61.90	59.60	65.30	65.10	62.30	69.70	55.00
(+) Likelihood ratio	1.620	1.545	1.741	1.465	1.366	1.741	1.670	1.507	1.967	1.398
(−) Likelihood ratio	0.006	0.008	0.606	0.714	0.752	0.606	0.641	0.693	0.580	0.675

WC: waist circumference; BMI: body mass index; WHtR: waist to height ratio; VAI: visceral adiposity index; ABSI: A body shape index; BRI: body roundness ind visceral adiposity index; CI: conicity index; TyG: triglyceride glucose; TyG-BMI: TyG related to BMI; TyG-WC: TyG related to WC; TyG-WHtR: TyG related to WHtR

## Data Availability

Data can be accessed via http://charls.pku.edu.cn/.

## Abbreviations

HTN: hypertension
BMI: body mass index
WC: waist circumference
WHtR: waist-height ratio
CI: conicity index
VAI: visceral adiposity index
CVAI: Chinese visceral adiposity index
LAP: lipid accumulation product
ABSI: a body shape index
BRI: body roundness index
TyG: triglyceride glucose
TyG-BMI: triglyceride glucose-body mass
TyG-WC: triglyceride glucose-waist circumference
TyG-WHtR: triglyceride glucose-waist-height ratio
ROC: receiver operator curve
AUC: area under curve
TG: triglyceride
HDL-C: high-density lipoprotein
BP: blood pressure
FPG: fasting plasma glucose blood glucose
CHARLS: China Health and Retirement Longitudinal Study
CAPI: computer-aided personal interview
OR: odds ratio
